# Genomic consequences of human‐mediated translocations in margin populations of an endangered amphibian

**DOI:** 10.1111/eva.13229

**Published:** 2021-03-25

**Authors:** Binia De Cahsan, Michael V. Westbury, Sofia Paraskevopoulou, Hauke Drews, Moritz Ott, Günter Gollmann, Ralph Tiedemann

**Affiliations:** ^1^ Unit of Evolutionary Biology/Systematic Zoology Institute for Biochemistry and Biology University of Potsdam Potsdam Germany; ^2^ GLOBE Institute University of Copenhagen Copenhagen Denmark; ^3^ Unit of Zoology Tel Aviv University Tel Aviv Israel; ^4^ Stiftung Naturschutz Schleswig‐Holstein Molfsee Germany; ^5^ Department of Evolutionary Biology University of Vienna Vienna Austria

**Keywords:** adaptive introgression, admixture, *Bombina bombina*, genetic rescue, mitogenomes, transcriptomics

## Abstract

Due to their isolated and often fragmented nature, range margin populations are especially vulnerable to rapid environmental change. To maintain genetic diversity and adaptive potential, gene flow from disjunct populations might therefore be crucial to their survival. Translocations are often proposed as a mitigation strategy to increase genetic diversity in threatened populations. However, this also includes the risk of losing locally adapted alleles through genetic swamping. Human‐mediated translocations of southern lineage specimens into northern German populations of the endangered European fire‐bellied toad (*Bombina bombina*) provide an unexpected experimental set‐up to test the genetic consequences of an intraspecific introgression from central population individuals into populations at the species range margin. Here, we utilize complete mitochondrial genomes and transcriptome nuclear data to reveal the full genetic extent of this translocation and the consequences it may have for these populations. We uncover signs of introgression in four out of the five northern populations investigated, including a number of introgressed alleles ubiquitous in all recipient populations, suggesting a possible adaptive advantage. Introgressed alleles dominate at the MTCH2 locus, associated with obesity/fat tissue in humans, and the DSP locus, essential for the proper development of epidermal skin in amphibians. Furthermore, we found loci where local alleles were retained in the introgressed populations, suggesting their relevance for local adaptation. Finally, comparisons of genetic diversity between introgressed and nonintrogressed northern German populations revealed an increase in genetic diversity in all German individuals belonging to introgressed populations, supporting the idea of a beneficial transfer of genetic variation from Austria into North Germany.

## 1 INTRODUCTION

Genetic admixture between populations is a widespread phenomenon that instantly increases the diversity of a population's gene pool and can enable a rapid adaptation to change in environmental conditions (Arnold & Kunte, 2017; Janes & Hamilton, 2017). However, in species with low dispersal abilities, natural gene flow may be limited among fragmented and isolated populations, especially those situated at the species' range margin. When facing rapid environmental changes, the adaptive potential at the margins may hence be compromised, as these genetically depauperate populations may suffer from genetic erosion and inbreeding (Arrendal et al., 2004; Madsen et al., 1999; Mussmann et al., 2017; Westemeier et al., 1998). Here, “genetic rescue” can be implemented as a mitigation strategy through translocation of individuals between populations (Johnson et al., 2010; Vilà et al., 2003). The transmission of beneficial gene variants from a closely related species or distant population is called “adaptive introgression” (Arnold & Kunte, 2017; Hedrick, 2013). Adaptive introgression could be particularly important in response to climate change as relevant alleles required at a species' poleward margin may already be present in more central populations. Introducing gene variants adapted to warmer conditions could potentially enhance the ability of the recipient poleward population to adapt more rapidly (Bridle & Vines, 2007).

One species with range margin populations that are particularly vulnerable is the fire‐bellied toad (*Bombina bombina*), a small amphibian species found in the Central and Eastern European lowlands. It mostly inhabits permanent, fish‐free freshwater bodies, like shallow stagnant lakes, ponds and swamps (Drews et al., 2011). After the Pleistocene, this species likely colonized Europe in two distinct expansions out of refugia located north‐west of the Black Sea (Fijarczyk et al., 2011; Hofman et al., 2007). The Carpathian Mountains and the Central European middle mountains at the border between Germany and the Czech Republic formed natural barriers that led to a north–south division of the species range. As a consequence, two genetically distinct evolutionary lineages of *B. bombina* emerged (Hofman et al., 2007).

Within the respective lineages, even geographically close populations of *B. bombina* are often genetically distinct, likely due to high site fidelity and limited dispersal capability (Dolgener et al., 2012; Engel, 1996; Schröder et al., 2012). This is particularly true for locally restricted and highly fragmented populations at the north‐western margin of the species range, which runs from the south of the Swedish province Scania to the Baltic Sea through Denmark and Germany (Schleswig‐Holstein, Niedersachsen, Sachsen‐Anhalt) (Schröder et al., 2012). In comparison with their counterparts in the central distribution range, margin populations of *B. bombina* are severely threatened by habitat alterations, pollution and intensive agriculture (Drews et al., 2011). Continuous human‐mediated habitat fragmentation has led to a reduction in gene flow and locally isolated populations are genetically depauperate (Schröder et al., 2012). *B. bombina* population sizes in Germany have been declining since the middle of the last century, which led to the species conservation status being listed as “critically endangered” within Germany (Kühnel et al., 2009).

Genetic analyses from toads collected in northern Germany in 2006 uncovered an introgression of southern lineage (putatively Austrian) genotypes into northern lineage populations. This was likely the result of occasional (and illegal) translocations of toads of southern origin into northern populations (Schröder et al., 2012). This study was, however, restricted to a short fragment of the mitochondrial Control Region and putatively neutral nuclear microsatellites, precluding inferences of the adaptive potential of these introgression events.

Here, we test the genetic impact of this translocation on the recipient populations on a genomic scale. Specifically, we searched entire mitochondrial genomes and nuclear transcriptomes for signs of introgression from southern lineage *B. bombina* from Austria into conspecific northern German *B. bombina* populations. We inferred levels of introgression in the nuclear genome and evaluated the resultant adaptive consequences by identifying introgressed alleles spreading in the northern German gene pool, as well as local northern German alleles retained, putatively due to local adaptation. We further investigated transcriptome‐wide genetic diversity of both introgressed and autochthonous populations.

## 2 MATERIALS AND METHODS

### 2.1 Samples

Thirty wild tadpoles were collected, five from Austria (Lobau, Vienna) in 2016 under permit MA22‐984143‐2015‐5 issued by the Municipality of Vienna and 25 from Germany (five each from Eutin/Röbel, Dannau, Högsdorf, Fehmarn and Neu‐Testorf) in 2017 under permits from Landesumweltamt Schleswig‐Holstein (Figure S1). German tadpoles were frozen in liquid nitrogen and stored at −80°C. The five Austrian tadpoles were transferred into a RNAlater storage solution (Sigma‐Aldrich Chemie GmbH).

### 2.2 RNA extraction, mRNA enrichment and cDNA library preparation

We extracted RNA from the tail muscle of the fins, using a modified Trizol isolation protocol in combination with a column‐based commercial kit (QIAGEN RNeasy Mini Kit) based on the manufacturer's protocol. Only skin and muscle tissue of the tadpole's fins were used in order to prevent contamination from gut bacteria during sequencing as much as possible. To further increase RNA yield, we transferred the frozen tadpole tails into a 1 ml Trizol solution and performed a homogenization with a Tissue Lyzer (6 min, 50 HZ). We implemented the column‐based clean‐up procedure from the QIAGEN Rneasy Mini Kit as described in the manufacturer's manual. We assessed the quality and quantity of the extracted total RNA on an Agilent Bioanalyzer 2100. We only selected samples with a high RIN number (>8) as well as a high RNA concentration (c > 50 ng/µl) for library preparation. We then performed an mRNA enrichment on these samples with poly(A) beads using the Bio Scientific NEXTflex Kit following the manufacturer's protocol. To assess the quantity of successfully enriched mRNA, we ran a Qubit High Sensitivity RNA Assay. We built cDNA libraries from the enriched mRNA samples with the NEXTflex Rapid Directional RNA‐Seq Kit (Bio Scientific) following the manufacturer's protocol. During the strand‐specific library preparation, we single indexed each sample and ran a PCR amplification with 15 cycles and an annealing temperature of 65°C. We performed a final quantity check on the built libraries with a dsDNA High Sensitivity Assay on the Qubit and a quality check on the Bioanalyzer. The cDNA library concentrations ranged from 2.94 to 17.4 ng/µl. We sent the libraries to be sequenced at Novogene in Hong Kong, China, where samples were additionally assessed for sufficient quantity and quality and PE 150 bp shotgun sequenced on three lanes on an Illumina HiSeq.

### 2.3 Quality filtering and nuclear mapping

We trimmed Illumina adapter sequences and poly A tails, only keeping reads with a quality score of at least 25 and a minimum length of 30 bp for all 30 samples using the software Skewer v0.2.2 (Jiang et al., 2014). We mapped the trimmed reads twice using Burrows–Wheeler algorithm (BWA) v0.7.15 (Li & Durbin, 2009) specifying the mem algorithm and default parameters to a previously published reference transcriptome of *B. bombina* from NCBI (GenBank accession: HADQ00000000.1) for population genomics analyses, and a transcriptome from *B. orientalis* (GenBank accession: HADT00000000.1) for use in downstream admixture analyses to avoid ascertainment bias that can be caused by mapping to an ingroup reference (Westbury et al., 2019), as well as a transcriptome from *Bombina variegata* (GenBank accession: HADR00000000.1). The resultant mapping files were then converted to BAM files and sorted using SAMtools v1.6.1 (Li & Durbin, 2009).

### 2.4 Mitogenomes

We additionally mapped the processed reads using BWA twice independently to two published reference *B. bombina* mitogenomes, one from Austria (GenBank accession: JX893173.1) (Pabijan et al., 2013) and the other from Germany (GenBank accession: MH893761.1) (De Cahsan et al., 2019). Due to insufficient and low read coverage, we removed one sample from the German Högsdorf population (Ge‐Ho‐KQ01). Mitochondrial consensus sequences were built using a maximum effective base depth approach in ANGSD (‐doFasta 3) (Korneliussen et al., 2014) and specifying the following parameters ‐minMapQ 25 ‐minQ 25 ‐uniqueOnly 1. We then compared the total number of mapped reads per individual for each reference (Table S1). In order to rule out ascertainment biases, which could be caused by mapping all individuals to a single reference from an ingroup population, we additionally constructed two independent maximum‐likelihood phylogenetic trees, one built using all individuals mapped to the Austrian reference and one using all individuals mapped to the German reference (Figure S2). This was done by computing an alignment of our 29 *B. bombina* samples and two outgroup mitogenome sequences (*B. orientalis* (GenBank accession: AY957562.1), *B. variegata* (GenBank accession: AY971143.1)) using MAFFT v7.392 (Katoh & Standley, 2013) and running RAxML v8.2.11 (Stamatakis, 2006, 2014), with 1000 bootstrap iterations, specifying a GTR+G substitution model. The resultant trees were then visually inspected for incongruencies. As the resultant trees were the same regardless of reference, we used the reference that had most reads mapping to it to construct a consensus sequence for each individual. These consensus sequences were aligned using MAFFT. We then produced an unrooted maximum‐likelihood phylogenetic tree from these aligned consensus sequences by running RAxML with 1000 bootstrap iterations, specifying a GTR+G substitution model.

### 2.5 Population transcriptomic analyses

We performed two independent principal component analyses with the software ANGSD v0.921 and PC*angsd* v0.95 (Meisner & Albrechtsen, 2018), one for all *B. bombina* individuals and one including only individuals from Germany. For both analyses, we used a minimum base quality (‐minQ) and a minimum mapping quality (‐minMapQ) parameter of 25; we required 20 individuals as the minimum number for a site to be considered for analysis (‐minInd 20), only included reads mapping uniquely to one location (‐uniqueonly), specified a minimum minor allele frequency of 0.05 (‐minFreq 0.05), called genotype likelihoods using the GATK method (‐GL 2), only included contigs over 1000 bp in size, required each site to have at least five reads mapping (‐mininddepth 5) and only considered SNPs with a *p*‐value lower than 1e‐6 (‐SNP_pval). The output genotype likelihoods were then converted from a beagle file into a covariance matrix using PCangsd. The same genotype likelihoods were also used to perform an admixture proportions analysis in PCangsd (‐admix). The most probable *K* value was chosen by the software based on the eigenvalues of the PCA.

### 2.6 Transcriptome‐wide *D*‐statistics/ABBA‐BABA analysis

We ran *D*‐statistics in ANGSD to look for the relative degree of gene flow in our samples. *D*‐statistics works based on a predefined species tree which uses three ingroup and one outgroup taxon. This topology can be written as (((H1,H2),H3),O) where H1 and H2 are more closely related to one another than either are to H3. *D*‐statistics then scans across the genome to find regions that break the known species tree, either due to incomplete lineage sorting or admixture between H3 and either H1 or H2. The occurrence of (((H1,H3),H2),O) being equal to (((H3,H2),H1),O), is interpreted as incomplete lineage sorting (or equal amounts of gene flow between H1+H3 and H2+H3). Sites at which H2 and H3 share a derived allele, while H1 and the outgroup share the ancestral allele, are called ABBA sites, while BABA represents sites at which H1 and H3 share the derived state and H2 and the outgroup share the ancestral allele. Any deviations from the expected 1:1 ratio are considered as differential levels of gene flow between the ingroup species analysed and are represented by the *D* value (null expectation of the test: *D* = 0). This value quantifies the deviation from the expected ratio, which is calculated by the differences in the sum of ABBA and BABA site patterns across the genome, divided by their sum:

D=[sum(ABBA)‐sum(BABA)]/[sum(ABBA)+sum(BABA)]

We used a random base sampling approach (‐doabbababa 1), considered only transcripts greater than 1000 bp in length and used the following filters: ‐minMapQ 25 ‐minQ 25 ‐uniqueOnly 1. As the sister species *B*. *variegata* is known to occasionally hybridize with *B. bombina* in our sampling area in north‐eastern Austria (Gollmann, 1984), we specified the related East Asian *B. orientalis* as outgroup. Because *B*. *orientalis* is more distantly related, it fits less well to the expectations of the infinite site model assumed by ABBA/BABA. The *D*‐statistics has, however, been found to be robust to small deviations from the infinite site model regarding the outgroup (Zheng & Janke, 2018). To investigate the significance of our result, we performed a weighted block jackknife test specifying a block size of a single contig. As is most commonly practised with determining the significance of *D*‐statistics, *D* values more than three standard errors different from zero (−3 < *Z *> 3) were considered as statistically significant, which corresponds to a *p*‐value <0.0013 (Green et al., 2010; Patterson et al., 2012; Zheng & Janke, 2018).

### 2.7 Transcriptome‐wide Treemix analysis

To further investigate the presence of admixture between Austrian and German lineages, we ran Treemix v1.1.3 (Pickrell & Pritchard, 2012) on the transcriptome data. To produce the input file for treemix, we first called pseudo haploid sequences using ANGSD, using the same parameters as for the PCA above with the addition of ‐doHaploCall 2 to call the consensus haploid base. This was done in a single run specifying all individuals from our study and the two outgroup individuals from NCBI (*B. variegata*, and *B. orientalis*). The output haploid file was converted to PLINK format using haplotoplink from the ANGSD toolsuite. We used plink v1.90b3.42 (Purcell et al., 2007) to convert the ANGSD output plink file into a Freq file, which was then converted into a treemix input file using plink2treemix.py (https://bitbucket.org/nygcresearch/treemix). We first ran treemix specifying no migration edges and *B. orientalis* as root to produce a consensus tree. We then used this tree as a prior when specifying multiple migration edges (‐m 1–4) while running treemix. The tree and residues were produced in R using the plotting_funcs.R script available with treemix (Figure 2a,b, and S5a‐h).

### 2.8 Genetic diversity

We estimated two independent measures of genetic diversity in our individuals, observed heterozygosity and nucleotide diversity. Heterozygosity gives an indication of the individual levels of diversity while the nucleotide diversity gives an indication of the population‐level diversity. We calculated heterozygosity using site allele frequencies determined using ANGSD. This was done independently for each individual using the same parameters as for the PCA with the addition of ‐doSaf 1. The resultant file was then run through realSFS from the ANGSD toolsuite with the parameter ‐nSites 50,000 to output the number of homozygous and heterozygous sites in windows of 50 kb of covered bases, traversing contig boundaries. To obtain population‐level estimates, we concatenated the results for all windows from all individuals within a single population.

For the nucleotide diversity estimates, we used the same pseudo haploid call file from the previous section. We then converted the output haploid call file into a geno file to be run through a suite of python scripts available online (https://github.com/simonhmartin/genomics_general). While running the popgenWindows.py, we specified the window size as 20 kb so that the window size encompasses complete contigs/genes, and a minimum number of sites per window as 100. The significance of differences between populations was calculated using a Wilcoxon–Mann–Whitney test while implementing a Bonferroni correction (Table S4).

### 2.9 Alleles highly differentiated between German and Austrian populations

The popgenWindows.py python script simultaneously calculated population differentiation statistics (*F*_st_) resulting in independent *F*_st_ values for each contig/transcript. From this output, we found contigs that were highly differentiated between the German and Austrian populations, in an attempt to uncover German/Austrian‐specific genes. We took the contigs showing the highest *F*_st_ values (top 5%) for each pairwise comparison of a German population against the Austrian population. This resulted in five pairwise comparisons (i.e. Austria–Fehmarn, Austria–Eutin, Austria–Dannau, Austria–Testorf and Austria–Högsdorf). We extracted the contigs that were found to be in the top 5% of all pairwise comparisons and cross‐referenced the sequences using BLAST to identify names and functions of these genes (Table S2 and S3).

### 2.10 Significantly introgressed alleles in the transcriptome

To find genes that exhibit a significant elevation of introgressed alleles in German populations, we ran the ABBABABAwindows.py (Martin et al., 2015) on the geno file from above, specifying *B. orientalis* as outgroup, all individuals from Austria as the H3 position, all individuals from Fehmarn as the H1 position and different combinations of the other German populations as H2. Fehmarn was chosen as H1, as it is considered autochthonous: It constitutes an island for which no introgression had been reported, physically separated from the other populations by the surrounding Baltic Sea. We specified the window size as 20 kb so that the window size encompasses complete contigs/genes, and a minimum number of sites per window as 100. This method computes the *F*_d_ statistic which is more robust to biases caused by genetic differentiation and nucleotide diversity when estimating *D* with small window sizes (Martin et al., 2015). We extracted the genes showing the strongest signs of admixture relative to Fehmarn (top 5%) and compared them between populations. From this, we estimated whether highly significant signs of admixture were found in the same contigs in all populations or whether they differed between populations, with prevalence of particular introgressed contigs in all introgressed populations putatively indicating a selective advantage. We used the online software tool panther (Protein ANalysis THrough Evolutionary Relationships) Classification System v14.1 ( Mi et al., 2013; Thomas et al., 2003) to assign genes with high levels of introgression to Gene Ontology classes.

## 3 RESULTS

### 3.1 Mitochondrial genomes

**FIGURE 1:**
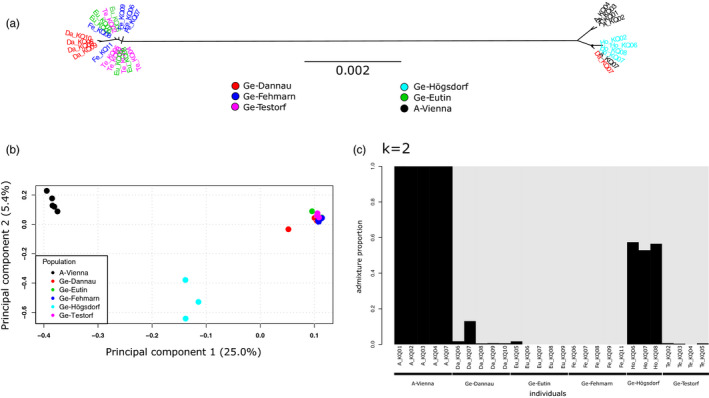
Population structure analyses (a) unrooted maximum‐likelihood tree for 29 mitochondrial genomes built with RAxML, (b) transcriptome‐wide principal component analysis of 27 *B. bombina* tadpole specimen from five locations in northern Germany and one location in Vienna, Austria, using the genotype‐likelihood method (GL), (c) Structure plot for 27 transcriptomes of German and Austrian *B. bombina* tadpoles (*k* = 2) from six different sampling locations

We obtained 30 complete mitochondrial genomes of *B. bombina* from tadpoles sampled in 2016 (Austria: Vienna) and 2017 (Germany: Fehmarn, Eutin/Röbel, Dannau, Högsdorf, Neu‐Testorf). A summary table of the mapping results is provided as Table S1. All Austrian individuals show a higher number of mapped reads when mapped to the Austrian reference genome. Four individuals from the German Högsdorf population, as well as one individual from Dannau (Ge‐Da‐KQ07), also contained higher read numbers mapping to the Austrian reference. All of the other German individuals from Dannau, Eutin, Fehmarn and Testorf contained more reads mapping to the German mitogenome reference than to the Austrian one. Regardless of mapping reference selected, phylogenetic trees were congruent in their topology confirming no bias in topology based on the chosen reference (Figure S2). The bootstrap values support the same two main clades in both trees. We then computed an unrooted tree using the consensus sequences produced by the reference which gave us the best mapping results for each individual. The resulting tree (Figure 1a) consists of two clades, one including all Austrian individuals, all four German Högsdorf individuals and one German Dannau individual (Ge‐Da‐KQ07). The other clade includes all German individuals from the populations Fehmarn, Eutin, Testorf and the remaining four individuals from Dannau. The distance between both clades (branch length/distance) shows a clear separation into a German and an Austrian haplogroup.

### 3.2 Nuclear mapping

When mapping our data to the reference transcriptome of *B. bombina*, we obtained an average read depth of 99.9 (min: 4.2, max: 158.5) and an average number of uniquely mapped reads of 40,467,551. When specifying *B. orientalis* as reference, the mapping resulted in an average read depth of 112.3 (min: 4.2, max: 177.8) and an average number of uniquely mapped reads of 37,805,633. Three samples, Ho_KQ001, Ho_KQ02 and Te_KQ01, were removed due to insufficient coverage (read depth <25). For more details, see Table S1.

### 3.3 Nuclear population structure

To examine whether the pattern found in the mitochondrial genomes is also reflected in the coding regions of the nuclear genome, the same cDNA libraries that we sequenced and used to reconstruct mitochondrial trees were also used to build transcriptomes for each individual. We ran a transcriptome‐wide principal component analysis (PCA) with the software ANGSD to identify population structure. For the analysis, we used a genotype‐likelihood (GL) approach (see Materials and methods). The resultant PCA groups all Austrian individuals into a single cluster and German individuals from Testorf, Eutin, Dannau and Fehmarn into a second cluster, in agreement with their geographical origin (Figure 1b). Interestingly, all individuals from Högsdorf form a separate third cluster. Moreover, one individual from the German Dannau sampling location (Ge‐Da‐KQ07) represents an outlier, although it exhibits some affinity to the remaining German individuals. This individual carries an Austrian mitochondrial haplotype (cf. Figure 1a). We ran an additional PCA on only the German populations to look for signs of substructure within our northern German sampling locations (Figure S3). The three German individuals from the population of Högsdorf are clearly separated from the other German localities and individual Ge‐Da‐KQ07 stands out even further from the German cluster (Fehmarn, Testorf, Eutin and Dannau). This result most likely reflects the high admixture proportion of introgressed Austrian alleles in the Högsdorf population. A scenario of recent admixture in Högsdorf is corroborated by the separation among the three Högsdorf individuals in the PCA (Figures 1 and S3), indicating that these specimens do not originate from a common gene pool in Hardy–Weinberg equilibrium, but rather each carries unique combinations of introgressed versus local alleles throughout their genomes.

### 3.4 Admixture analyses

In combination, our findings based on the mitochondrial genomes and the PCA on nuclear data suggest admixture between Austrian and some German populations. To identify the degree of admixture between southern (Austria) and northern (Germany) European fire‐bellied toads, we performed an admixture structure analysis using PCangsd (Meisner & Albrechtsen, 2018). The admixture plot for *K* = 2 shows a high level of shared alleles between all German Högsdorf individuals and Austrian toads; that is, approximately half of the genetic information coming from transcriptome‐wide SNPs are shared with the Austrian individuals (Figure 1c). We also found small levels of Austrian introgression in all other German sampling locations except for the island of Fehmarn, where all sampled individuals appear to be uniform, presumably resembling an autochthonous German population. The second highest level of shared alleles between Austrian and German toads was found in individuals from Dannau, most notably in the tadpole Ge‐Da‐KQ07. These results are congruent with the results from the PCAs and the mitochondrial data.

**FIGURE 2:**
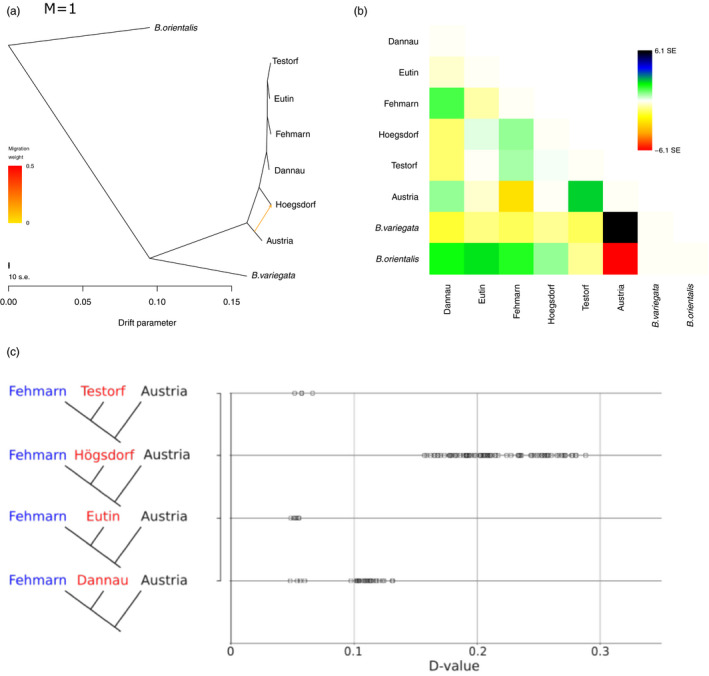
Tests for admixture between Austrian and northern German tadpoles (a) treemix tree for a single migration edge. *B. orientalis* is specified as root, (b) residue matrix for the treemix tree with *m* = 1, (c) ABBA‐BABA or *D*‐statistics analysis for 27 transcriptomes of German and Austrian *B. bombina* tadpoles, outgroup in all analyses is *B. orientalis* (c) *D* values for H1 = Fehmarn, H2 = Testorf/Högsdorf/Eutin/Dannau, H3 = Austria

To further evaluate the relative level of introgression between the Austrian (southern lineage) and German (northern lineage) populations, we carried out an ABBA‐BABA or *D*‐statistics analysis for all 27 samples. Among the German populations, ancestral population structure is, however, unlikely as northern *B. bombina* populations are assumed to originate from a single panmictic population that had postglacially split from the southern lineage (Hofman et al., 2007). All resulting comparisons with an insignificant *Z* score (a value between −3 and 3) were removed from further analyses. As previous analyses indicated toads from Fehmarn not to carry Austrian alleles, we considered the Fehmarn population to represent the autochthonous (nonintrogressed) condition. Consequently, we plotted all significant *D* values for the remaining four German sampling locations, while constraining Fehmarn to the H1 branch and Austria to the H3 (Figure 2c). A positive *D* value indicates admixture between the H2 and the H3 population, while a negative *D* value indicates admixture between the H1 and the H3 population.

For all four German populations, we found positive *D* values indicating higher levels of admixture between the Austrian population and the German populations from Testorf, Högsdorf, Eutin and Dannau compared to Fehmarn, ranging from 0.048 to 0.288. The German Högsdorf population had the highest *D* values from 0.157 to 0.288, followed by Dannau with 0.144 to 0.194. The Testorf and Eutin populations show similar positive, low *D* values (around 0.05), indicating a smaller degree of admixture with Austrian toads in these populations.

Our treemix analyses also confirm the result that the Högsdorf population is admixed with Austrian toads. When specifying no migration edges, we obtained the expected tree topology with all German populations being sister to the Austrian population. The residue matrix for this tree had a high SE between Högsdorf and Austria indicating that a migration edge between the two may be missing. We therefore ran treemix with 1, 2, 3 and 4 migration edges. When specifying a single migration edge, as expected due to the residue matrix of *m *= 0, we uncover migration between Austria and Högsdorf (Figure 2a,b). However, when specifying more than one migration edge, the tree topology changes and Austria and Högsdorf become a sister clade to the remaining German populations. This is likely due to the high admixture proportion of Austrian alleles in Högsdorf.

### 3.5 Genetic diversity

**FIGURE 3:**
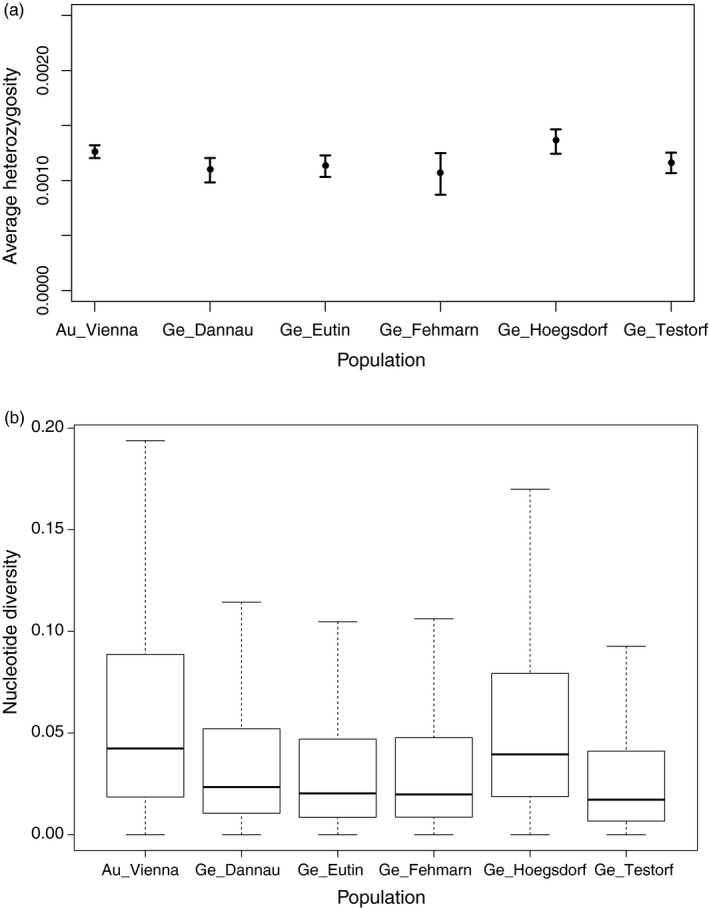
Genetic diversity across the transcriptome (a) comparison of transcriptome‐wide heterozygosity among populations. (b) Comparison of transcriptome‐wide nucleotide diversity (pi) among populations

We independently calculated observed heterozygosity for all *B. bombina* individuals in this study (Figure 3a). We found the highest average level of heterozygosity in individuals from the German population of Högsdorf and in the Austrian toads. The nucleotide diversity (pi) follows a similar pattern (Figure 3b). When considering both heterozygosity and pi, we found the difference in diversity levels between Högsdorf and Austria to be nonsignificant, while these two populations had significant differences to the remaining four German populations (Table S4).

### 3.6 Highly differentiated genes between German and Austrian populations

Using the population differentiation statistics (*F*_st_), we were able to identify loci that were highly differentiated (top 5%) between the German and Austrian populations. We uncovered 23 contigs found to be highly differentiated in all pairwise comparisons between the German and Austrian populations. We cross‐referenced these contigs via BLAST and identified gene names for 17 of the 23 genes of interest (Table S2). All 17 identified loci belong to genes that appear to play an important role for the general functioning of cells. However, one gene (FAM3A) is additionally associated with antifungal properties.

### 3.7 Genes showing high levels of introgression

**FIGURE 4:**
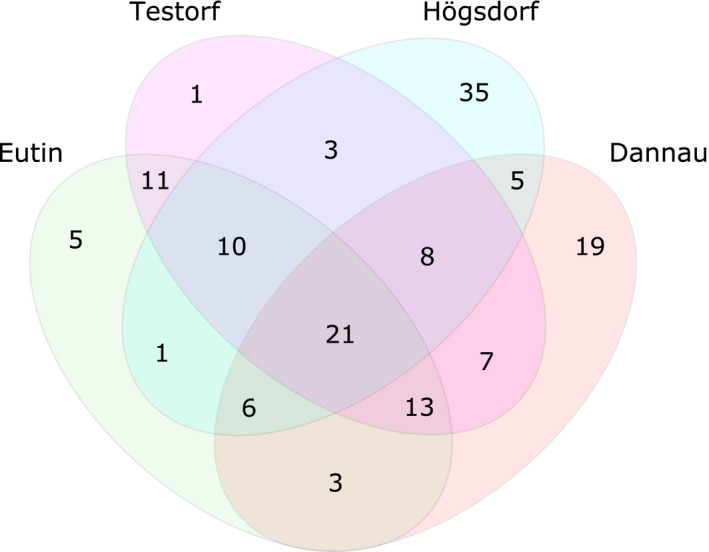
VENN diagram of the four introgressed German populations. The numbers represent shared and unique admixed loci, which showed the strongest signs of admixture with Austria relative to Fehmarn (top 5% of *F*_d_ values), within and among populations (putative candidate genes for adaptive introgression)

To uncover which introgressed alleles may be adaptive, we utilized *F*_d_ statistics (Martin et al., 2015). We uncovered a number of genes showing the strongest signs of admixture (top 5% *F*_d_) relative to Fehmarn (considered autochthonous, see above) in the remaining German populations (Figure 4). We identified 21 loci, showing consistent strong signals of admixture in all four German populations, excluding Fehmarn. We successfully found matches for eight out of the 21 loci using BLAST and were able to classify five of them (Table S3 and Figure S6). Interestingly, two of these genes are known to be involved in immune and stress response‐related processes, as well as metabolic pathways. The Mitochondrial Carrier 2 gene (MTCH2) is highly expressed in white adipose tissue and adipocytes and thought to play a regulatory role in adipocyte differentiation. The desmoplakin (DSP) gene encodes a unique and vital desmosomal protein that is integral to epidermal development. Further analyses into amino acid changes of these two genes showed no obvious amino acid changes consistent with gene flow between northern and southern populations (i.e. different amino acid in Fehmarn compared to all other populations).

## 4 DISCUSSION

The question of whether the benefits of translocations as a method for genetic rescue outweigh the downfalls is a highly relevant topic as many species now require at least some means of human‐mediated intervention for their survival (Barton, 2001; Mansfield & Land, 2002; Seddon, 2010). We sought to address this question through the production of complete mitochondrial genomes and transcriptomes from an endangered amphibian species, the European fire‐bellied toad. For this, we sampled five endangered northern *B. bombina* populations located in Germany and one southern *B*. *bombina* population from Austria and assessed the genetic consequences of human‐mediated translocations of Austrian *B. bombina* populations into the range margin northern German populations.

We confirmed the presence of southern “alien” (i.e. Austrian) DNA in the northern populations as was previously found using toads collected in 2006 (Schröder et al., 2012). However, in contrast to the previous study where the introgression was found limited to one population (Högsdorf), all of our German sampling sites, except for Fehmarn (an island population separated from the mainland by the brackish water of the Baltic Sea) showed traces of introgression from the South (Figure 2c). This increase in the presence of signs of introgression could have a number of explanations. One simple explanation for this could be the increase in number and sensitivity of the chosen marker or pure stochastic chance during sampling. Another scenario is migration of introgressed *B. bombina* individuals between sites over the last 10 years, spreading the introgressed alleles to neighbouring populations. The latter appears probable as adult toads regularly migrate up to 500 m and occasionally more than 1 km over a period of multiple days (Engel, 1996; Günther & Schneeweiss, 1996). Closely related *B. variegata* have been shown to migrate up to 90 m per night (Abbühl & Durrer, 1996).

Next, to uncover whether this introgression may have had adaptive consequences, we investigated which specific Austrian alleles were introgressed into the northern German populations and how widespread they became among the German populations. We found a number of gene variants present in all of the four admixed German populations indicating that they may have some selective advantage to the recipient toads leading to their retention (Figure 4 and Table S3). Alternatively, these gene variants could have become locally abundant due to random effects (i.e. genetic drift) and subsequently spread by dispersal. However, in favour of a potential selective advantage of the introgression event(s), two of these genes (MTCH2, and DSP) display functions that could putatively be linked to differential fitnesses. The MTCH2 gene is highly expressed in white adipose tissue (Kulyté et al., 2011; Speliotes et al., 2010) and has been linked to obesity in humans through genomewide association and expression studies (Kulyté et al., 2011; Speliotes et al., 2010). The Austrian MTCH2 gene variant may represent an adaptive advantage for northern German *B. bombina* toads as it could influence the storage efficiency of fat as a form of energy. In amphibians, a greater body mass often correlates with higher reproductive success (Feder & Burggren, 1992; Kuramoto, 1978; MacCracken & Stebbings, 2012). DSP encodes a unique desmosomal protein vital for epidermal development (Garrod & Chidgey, 2008) and has been shown to play a critical role in *Xenopus laevis* embryos during the frog's epidermal morphogenesis (Bharathan & Dickinson, 2019). DSP appears to be required to move basally located cells into the outer epidermal layer, where they differentiate into specific epidermal cell types and are fundamental for the skin's integrity (Bharathan & Dickinson, 2019). An intact and resilient skin is vital for amphibians, especially at the tadpole stage as it protects them from pathogens through secretion as well as from mechanical stress. A reduction or lack of DSP in *X*. *laevis* embryos led to skin fragility and reduced resistance to mechanical stress (Bharathan & Dickinson, 2019). Although we did not detect amino acid changes within these two genes consistent with gene flow between northern and southern *B. bombina* populations, upstream cis‐regulatory elements were likely also introgressed due to linkage disequilibrium which may influence function. However, we are unable to look for lineage‐specific cis‐regulatory elements using the dataset at hand.

Despite the presence of these putatively beneficial introgressed alleles, the question remains in how far genetic swamping may have compromised local adaptation in these introgressed populations, putatively reducing the fitness of admixed individuals. Therefore, we also searched for genes where local northern German alleles may have been retained despite admixture, potentially due to a role in adaptation to the local environment. We found that most such genes play an essential role for the general functioning of cells and therefore might be crucial for the German toads. However, one gene (FAM3A) stood out as it encodes for a peptide known as Drosomycin‐Like Defensin (DLD). Studies on humans and *Drosophila melanogaster* discovered that DLD may act as a defence against invading fungal microorganisms (Simon et al., 2008). It seems reasonable to assume that this antifungal activity may be a sign of German toads having evolved a defence mechanism against the local fungal pathogens, as this allele remains present in the northern German gene pool despite ongoing admixture. This is in line with the finding of a previous study that local alleles are retained in genes of the adaptive immune system (MHC), despite introgression (Schröder et al., 2012).

The presence of introgressed potentially beneficial alleles at some genes, but local alleles at other genes in the recipient northern German populations could constitute the result of natural selection, leading to mosaic genotypes with favourable alleles at multiple loci, be they introgressed or autochthonous. We further found that the introduction of allochthonous individuals led to an increase in genetic diversity of the recipient populations (Figure 3). This increase in genetic diversity may provide a new lifeline for the endangered range margin populations of this species as they are often highly fragmented, have low levels of genetic diversity, and lack gene flow from surrounding populations, meaning they have little chance of rapidly accumulating new alleles naturally (Arnaud‐Haond et al., 2006; Bridle & Vines, 2007). Although the translocation of individuals between populations can be risky to recipient populations, by introducing foreign parasites or pathogens (Cunningham, 1996; Kock et al., 2010) or disrupting locally adapted gene assemblages via an influx of foreign alleles (Edmands, 2007), it can also be beneficial. Adaptive introgression can instantly increase the genetic diversity of depauperate populations by providing new adaptive gene variants and reducing the level of inbreeding and therefore the chance of rare deleterious alleles being present in a homozygous state (Charlesworth & Willis, 2009; Edmands, 2007) which may be the case in fragmented northern fire‐bellied toad populations. We show that the translocation of individuals from Austria had a significant genetic impact on northern German populations, with a tendency of spreading further. We find evidence that the introduction of alleles from Austria into Germany may have been beneficial to the recipient populations through the increase in genetic diversity and introduction of new alleles that were subsequently retained at high frequencies. Moreover, positive population trends have been reported for these introgressed populations (Drews et al., 2011), further corroborating our findings and our hypothesis that the introgression of southern alleles had a positive effect on the endangered northern populations.

## 5 CONCLUSIONS

Through the production of mitochondrial genomes and transcriptomic data from six populations of *B. bombina*, we were able to assess the genetic consequences of a human‐mediated translocation of individuals from the more genetically robust central Austrian population into endangered range margin populations in northern Germany. Although conservationists may be reluctant to translocating individuals from different evolutionary lineages as means of genetic rescue (Kock et al., 2010; Schröder et al., 2012), we show that this introgression may have been beneficial to the recipient populations by introducing new alleles at some loci, while maintaining locally adaptive alleles at others, overall increasing the genetic diversity of the population. Unless local populations are completely swamped by allochthonous individuals, crossbreeding between allochthonous and autochthonous individuals—as evidenced in our study—can provide ample diversity in allele combinations, upon which natural selection can act. Our results provide important insights into how wild populations may genetically benefit from human‐mediated translocations that will undoubtedly become increasingly necessary as more and more species face elevated extinction risks due to human activity and global climatic changes.

## CONFLICT OF INTEREST

The authors declare that they have no conflict of interest.

## Supporting information

Supplementary Material

## Data Availability

The data that support the findings of this study are openly available on GenBank at https://www.ncbi.nlm.nih.gov/genbank/, accession numbers MW646490–MW646518 (mitogenomes) and BioProject number PRJNA702749 (transcriptome raw data).
