# Supplementary material for: Genomic consequences of human‐mediated translocations in margin populations of an endangered amphibian
Source: Evol Appl. 2021 Mar 25;14(6):1623–34. doi: 10.1111/eva.13229 (PMC8210794; doi:10.1111/eva.13229)
Supplement: Supplementary file 1 — Supplementary Material [file EVA-14-1623-s001.docx]

**Supplementary Material**

**Tables**

**Table S1:** Comparison of total number of mapped reads for each tadpole sample and one *B. orientalis* specimen to either a German (Genbank Accession: MH893761.1) or an Austrian reference mitogenome (Genbank Accession: JX893173.1) and comparison of total number of uniquely mapped reads and average read depth (coverage) for each tadpole sample and one *B. orientalis* specimen to either the German (Genbank Accession: HADQ00000000.1) or *B. orientalis* reference transcriptome (Genbank Accession: HADT00000000.1).The three samples marked with an asterisk were excluded from downstream analysis due to low read coverage.


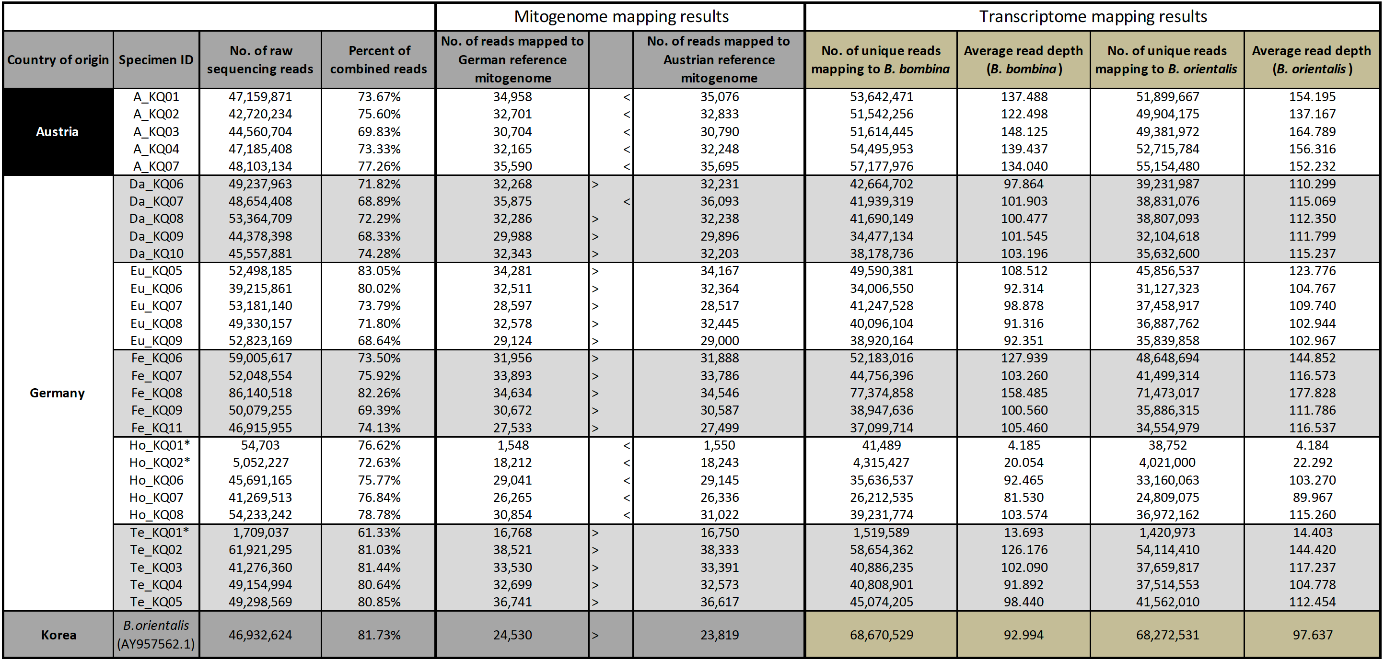


**Table S2:** List of identified genes that may be retained in northern German populations according to population differentiation statistics (Fst)

| **#** | **Contig name** | **Gene (BLAST)** | **Function** (ENTREZ/GeneCards/UniProt Gene Summary) |
| --- | --- | --- | --- |
| 1 | HADT01000147.1 | *No significant similarity found* |  |
| 2 | HADT01007038.1 | Xenopus tropicalis ATPase, H+ transporting, lysosomal 31kDa, V1 subunit E1 (atp6v1e1) | This gene encodes a component of vacuolar ATPase (V-ATPase), a multisubunit enzyme that mediates acidification of eukaryotic intracellular organelles. Mutant phenotypes in humans show i.a. abnormal apolipoprotein levels and abnormal subcutaneous fat tissue distribution. The V1 domain consists of three A, three B, and two G subunits, as well as a C, D, E, F, and H subunit. The V1 domain contains the ATP catalytic site. This gene encodes alternate transcriptional splice variants, encoding different V1 domain E subunit isoforms. |
| 3 | HADT01009594.1 | Mustela putorius furo solute carrier family 36, member 1 (SLC36A1) | This gene encodes a member of the eukaryote-specific amino acid/auxin permease (AAAP) 1 transporter family. The encoded protein functions as a proton-dependent, small amino acid transporter. Associated phenotypes from GWAS in humans are atopic eczema and psoriasis. |
| 4 | HADT01009935.1 | Crocodylus porosus interferon regulatory factor 2 (IRF2) | IRF2 encodes interferon regulatory factor 2, a member of the interferon regulatory transcription factor (IRF) family. IRF2 competitively inhibits the IRF1-mediated transcriptional activation of interferons alpha and beta, and presumably other genes that employ IRF1 for transcription activation. However, IRF2 also functions as a transcriptional activator of histone H4. |
| 5 | HADT01010756.1 | Fulmarus glacialis aminoadipate aminotransferase (AADAT) | This gene encodes a protein that is highly similar to mouse and rat kynurenine aminotransferase II. One activity is the transamination of alpha-aminoadipic acid, a final step in the saccaropine pathway which is the major pathway for L-lysine catabolism. The other activity involves the transamination of kynurenine to produce kynurenine acid, the precursor of kynurenic acid which has neuroprotective properties. |
| 6 | HADT01016829.1 | *No significant similarity found* |  |
| 7 | HADT01017495.1 | Chiroxiphia lanceolata ubiquitin specific peptidase 24 (USP24) | Ubiquitin-specific protease that regulates cell survival in various contexts through modulating the protein stability of some of its substrates including DDB2, MCL1 or TP53. Plays a positive role on ferritinophagy where ferritin is degraded in lysosomes and releases free iron. |
| 8 | HADT01018942.1 | *No significant similarity found* |  |
| 9 | HADT01021783.1 | Xenopus tropicalis membrane protein, palmitoylated 1 (mpp1) | This gene encodes the prototype of the membrane-associated guanylate kinase (MAGUK) family proteins. MAGUKs interact with the cytoskeleton and regulate cell proliferation, signaling pathways, and intercellular junctions. This gene product interacts with various cytoskeletal proteins and cell junctional proteins in different tissue and cell types and may be involved in the regulation of cell shape, hair cell development, neural patterning of the retina, and apico-basal polarity and tumor suppression pathways in non-erythroid cells. Human phenotypes associated with mpp1 are hemoglobin and erythrocyte count. |
| 10 | HADT01022144.1 | Xenopus tropicalis von Hippel-Lindau binding protein 1 (vbp1) | The protein encoded by this gene interacts with the Von Hippel-Lindau protein to form an intracellular complex. The encoded protein functions as a chaperone protein and may play a role in the transport of the Von Hippel-Lindau protein from the perinuclear granules to the nucleus or cytoplasm. |
| 11 | HADT01022313.1 | Xenopus laevis translocase of inner mitochondrial membrane 17A S homeolog (timm17a.S) | Essential component of the TIM23 complex, a complex that mediates the translocation of transit peptide-containing proteins across the mitochondrial inner membrane. |
| 12 | HADT01023024.1 | Strigops habroptila solute carrier family 26 member 11 (SLC26A11) | This gene encodes a member of the solute linked carrier 26 family of anion exchangers. Members of this family of proteins are essential for numerous cellular functions including homeostasis and intracellular electrolyte balance. Among its related pathways are Transport of glucose and other sugars, bile salts and organic acids, metal ions and amine compounds. |
| 13 | HADT01023193.1 | Xenopus laevis enolase 3 (beta, muscle) L homeolog (eno3.L) | This gene encodes one of the three enolase isoenzymes found in mammals. This isoenzyme is found in skeletal muscle cells in the adult where it may play a role in muscle development and regeneration. A switch from alpha enolase to beta enolase occurs in muscle tissue during development in rodents. Mutations in this gene have be associated glycogen storage disease. |
| 14 | HADT01024783.1 | Xenopus laevis WASH complex subunit strumpellin (LOC108695443) | The WASH (WASP and Scar homologue) complex is present at the surface of endosomes and recruits and activates the Arp2/3 complex to induce mediated actin nucleation. The WASH complex plays a key role in the fission of tubules that serve as transport intermediates during endosome sorting. |
| 15 | HADT01026945.1 | Xenopus tropicalis UPF1 regulator of nonsense transcripts homolog (upf1) | This gene encodes a protein that is part of a post-splicing multiprotein complex involved in both mRNA nuclear export and mRNA surveillance. |
| 16 | HADT01029441.1 | *No significant similarity found* |  |
| 17 | HADT01030418.1 | Xenopus laevis coenzyme Q8A L homeolog (coq8a.L) | This gene encodes a mitochondrial protein similar to yeast ABC1, which functions in an electron-transferring membrane protein complex in the respiratory chain. |
| 18 | HADT01030582.1 | Xenopus tropicalis MOB kinase activator 1A (mob1a) | The protein encoded by this gene is a component of the Hippo signaling pathway, which controls organ size and tumor growth by enhancing apoptosis. Loss of the encoded protein results in cell proliferation and cancer formation. |
| 19 | HADT01030774.1 | *No significant similarity found* |  |
| 20 | HADT01032041.1 | Nanorana parkeri family with sequence similarity 3 member A (FAM3A) | This gene encodes a cytokine-like protein. The expression of this gene may be regulated by peroxisome proliferator-activated receptor gamma, and the encoded protein may be involved in the regulation of glucose and lipid metabolism. May act as a defensin against invading fungal microorganisms. |
| 21 | HADT01032731.1 | *No significant similarity found* |  |
| 22 | HADT01034986.1 | Salmo salar probable ATP-dependent RNA helicase (DDX17) | DEAD box proteins, characterized by the conserved motif Asp-Glu-Ala-Asp (DEAD), are putative RNA helicases. They are implicated in a number of cellular processes involving alteration of RNA secondary structure, such as translation initiation, nuclear and mitochondrial splicing, and ribosome and splicesosome assembly. Based on their distribution patterns, some members of this family are believed to be involved in embryogenesis, spermatogenesis, and cellular growth and division |
| 23 | HADT01056253.1 | Xenopus laevis peroxisome proliferator activated receptor alpha L homeolog (ppara.L) | Ligand-activated transcription factor. Key regulator of lipid metabolism. Receptor for peroxisome proliferators such as hypolipidemic drugs and fatty acids. Regulates the peroxisomal beta-oxidation pathway of fatty acids. Functions as transcription activator for the ACOX1 and P450 genes. |

**Table S3:** List of identified genes, which are highly admixed according to Fd statistics between the putatively unadmixed Fehmarn and Austrian individuals and present in all four admixed German populations Eutin, Dannau, Testorf and Högsdorf

| **#** | **Contig name** | **Gene (BLAST)** | **Function** (ENTREZ/GeneCards/UniProt Gene Summary) |
| --- | --- | --- | --- |
| 1 | HADT01019260.1 | Alligator sinensis Snf2 related CREBBP activator protein (SRCAP) | This gene encodes the core catalytic component of the multiprotein chromatin-remodeling SRCAP complex. The encoded protein is an ATPase that is necessary for the incorporation of the histone variant H2A.Z into nucleosomes. It can function as a transcriptional activator in Notch-mediated, CREB-mediated and steroid receptor-mediated transcription. Mutations in this gene cause Floating-Harbor syndrome, a rare disorder characterized by short stature, language deficits and dysmorphic facial features in humans. Associated phenotypes are erythrocyte count, corpuscular hemoglobin and corpuscular volume. |
| 2 | HADT01020975.1 | Xenopus tropicalis sphingomyelin phosphodiesterase 1 (smpd1) | The protein encoded by this gene is a lysosomal acid sphingomyelinase that converts sphingomyelin to ceramide. The encoded protein also has phospholipase C activity. Defects in this gene are a cause of Niemann-Pick, an inherited disease in humans that affects the body's ability to metabolize fat (cholesterol and lipids) within cells. Abnormal blood gas levels and abnormal bleeding are mutant phenotypes linked to smpd1. |
| 3 | HADT01027065.1 | Xenopus tropicalis widely interspaced zinc finger motifs (wiz) | WIZ (WIZ Zinc Finger) is a Protein Coding gene. Diseases associated with WIZ include Exstrophy Of Bladder and Bladder Exstrophy-Epispadias-Cloacal Exstrophy Complex. Gene Ontology (GO) annotations related to this gene include SET domain binding. Phenotypes associated with this gene include platelet count and volume. |
| 4 | HADT01027139.1 | *No significant similarity found* |  |
| 5 | HADT01027162.1 | *No significant similarity found* |  |
| 6 | HADT01027814.1 | Nanorana parkeri mitochondrial carrier 2 (MTCH2) | Encodes a member of the SLC25 family of nuclear-encoded transporters that are localized in the inner mitochondrial membrane. Members of this superfamily are involved in many metabolic pathways and cell functions. Genome-wide association studies in human have identified single-nucleotide polymorphisms in several loci associated with obesity. This gene is one such locus, which is highly expressed in white adipose tissue and adipocytes and thought to play a regulatory role in adipocyte differentiation and biology and is i.a. phenotypically associated with body fat distribution and systolic blood pressure. |
| 7 | HADT01027837.1 | No significant similarity found |  |
| 8 | HADT01029582.1 | Esox lucius isolate pG10E-44 transposon Tc1-like | Transposon |
| 9 | HADT01029691.1 | *No significant similarity found* |  |
| 10 | HADT01030419.1 | *No significant similarity found* |  |
| 11 | HADT01030616.1 | *No significant similarity found* |  |
| 12 | HADT01030754.1 | *No significant similarity found* |  |
| 13 | HADT01031481.1 | Xenopus tropicalis desmoplakin (dsp), misc_RNA | This gene encodes a protein that anchors intermediate filaments to desmosomal plaques and forms an obligate component of functional desmosomes. Mutations in this gene are the cause of several cardiomyopathies and keratodermas, including skin fragility-woolly hair syndrome. Dsp linked phenotypes in humans include abnormalities in hair, nail and the cardiovascular system as well as a loss of intercellular connections, such as desmosomes, resulting in loss of cohesion between keratinocytes. |
| 14 | HADT01031696.1 | Xenopus laevis ATPase H+ transporting V1 subunit A L homeolog (atp6v1a.L) | This gene encodes a component of vacuolar ATPase (V-ATPase), a multisubunit enzyme that mediates acidification of eukaryotic intracellular organelles. Mutant phenotypes in humans show i.a. abnormal apolipoprotein levels and abnormal subcutaneous  fat tissue distribution. |
| 15 | HADT01032080.1 | *No significant similarity found* |  |
| 16 | HADT01032434.1 | Xenopus laevis GLI pathogenesis-related 2 L homeolog (glipr2.L) | GLIPR2 (GLI Pathogenesis Related 2) is a Protein Coding gene. Gene Ontology (GO) annotations related to this gene include protein homodimerization activity |
| 17 | HADT01033789.1 | *No significant similarity found* |  |
| 18 | HADT01034420.1 | *No significant similarity found* |  |
| 19 | HADT01034651.1 | *No significant similarity found* |  |
| 20 | HADT01035036.1 | *No significant similarity found* |  |
| 21 | HADT01035493.1 | *No significant similarity found* |  |

**Table S4:** Bonferroni corrected p-values retrieved from Wilcoxon Mann Whitney test for genetic diversity (Nucleotide diversity and observed heterozygosity) comparisons between all six populations. Au - Austria; GE_Da - Germany, Dannau; GE_Fe - Germany, Fehmarn; GE_Te – Germany, Testorf; GE_Ho – Germany, Högsdorf; GE_Eu – Germany, Eutin

| **Nucleotide diversity (pi)** | pi(AU) | pi(GE_Da) | pi(GE_Fe) | pi(GE_Te) | pi(GE_Ho) |
| --- | --- | --- | --- | --- | --- |
| pi(GE_Da) | <3,30E-15 |  |  |  |  |
| pi(GE_Fe) | <3,30E-15 | 6,79E-09 |  |  |  |
| pi(GE_Te) | <3,30E-15 | <3,30E-15 | <3,30E-15 |  |  |
| pi(GE_Ho) | 1,00 | <3,30E-15 | <3,30E-15 | <3,30E-15 |  |
| pi(GE_Eu) | <3,30E-15 | 8,08E-04 | 0,38 | <3,30E-15 | <3,30E-15 |
|  |  |  |  |  |  |
| **Heterozygosity (het)** | het(AU) | het (GE_Da) | het(GE_Fe) | het(GE_Te) | het(GE_Ho) |
| het(GE_Da) | <3,30E-15 |  |  |  |  |
| het(GE_Fe) | <3,30E-15 | 1,000 |  |  |  |
| het(GE_Te) | <3,30E-15 | 1,000 | 1,000 |  |  |
| het(GE_Ho) | 1,000 | <3,30E-15 | <3,30E-15 | <3,30E-15 |  |
| het(GE_Eu) | <3,30E-15 | 0,877 | 0,055 | 0,189 | <3,30E-15 |

**Figures**

**
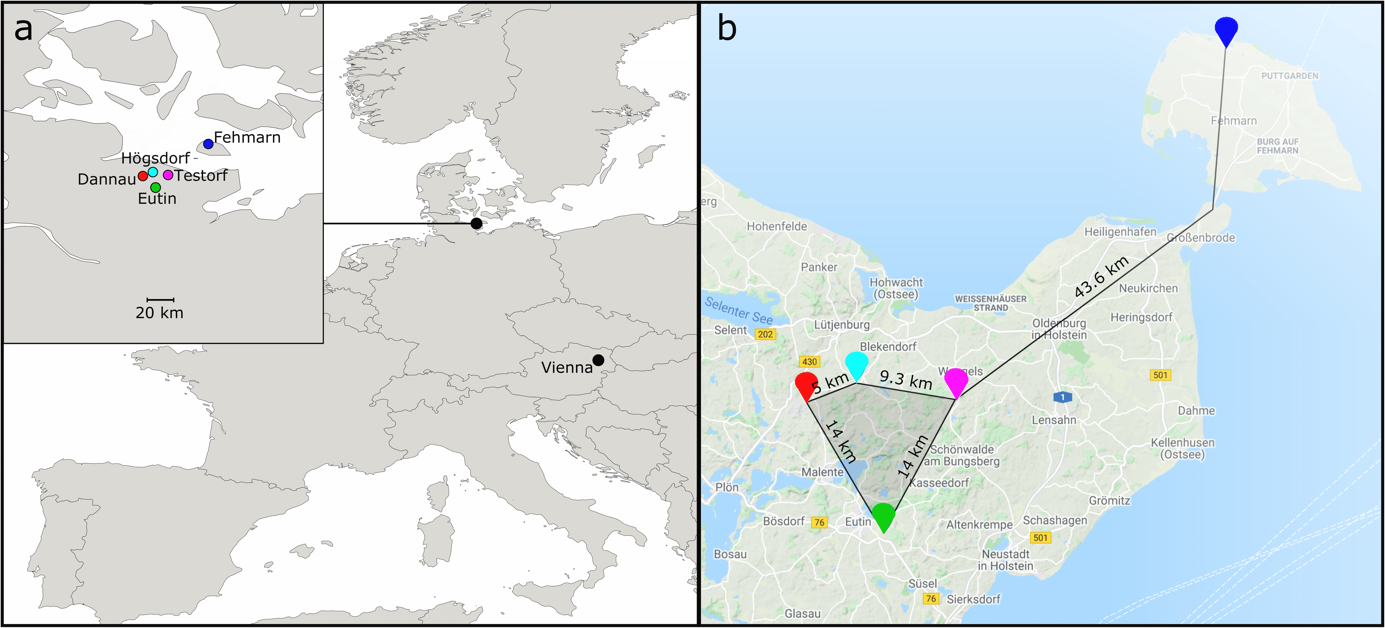
**

**Figure S1:** Map of sampling locations in a) Germany (n=5) and Austria (n=1). b) Approximate migration distances between the five sites in northern Germany. Note that the island of Fehmarn is connected to the mainland by a 963m long bridge only.

**A**


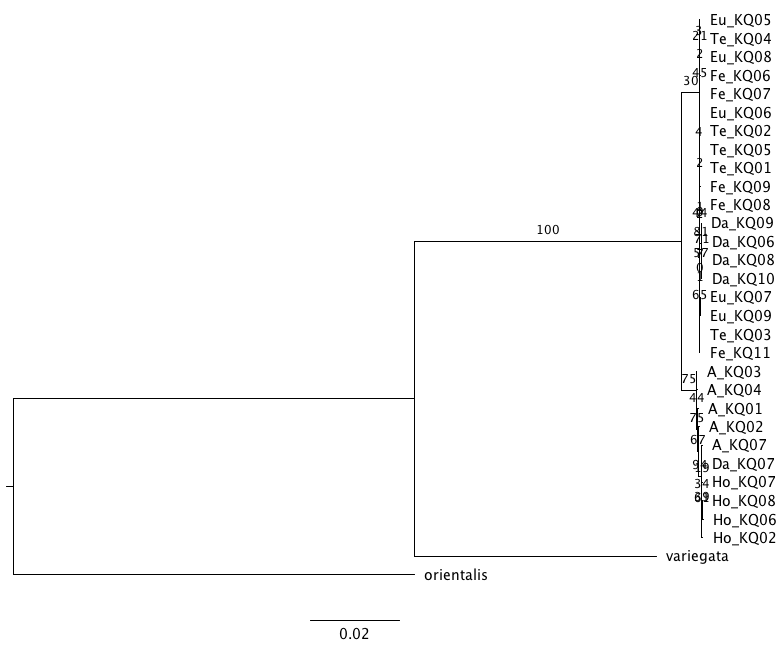


**B**


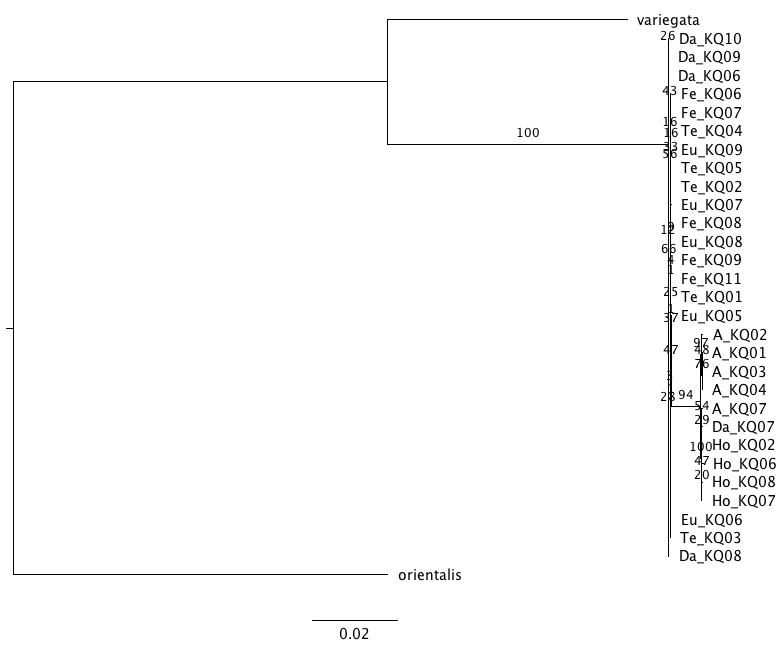


**Figure S2:** Maximum Likelihood trees of 29 mitogenomes mapped to (A) a German reference mitogenome or (B) an Austrian reference mitogenome using RaxML specifying *B. orientalis* as outgroup. Numbers on branch lengths represent bootstrap values.


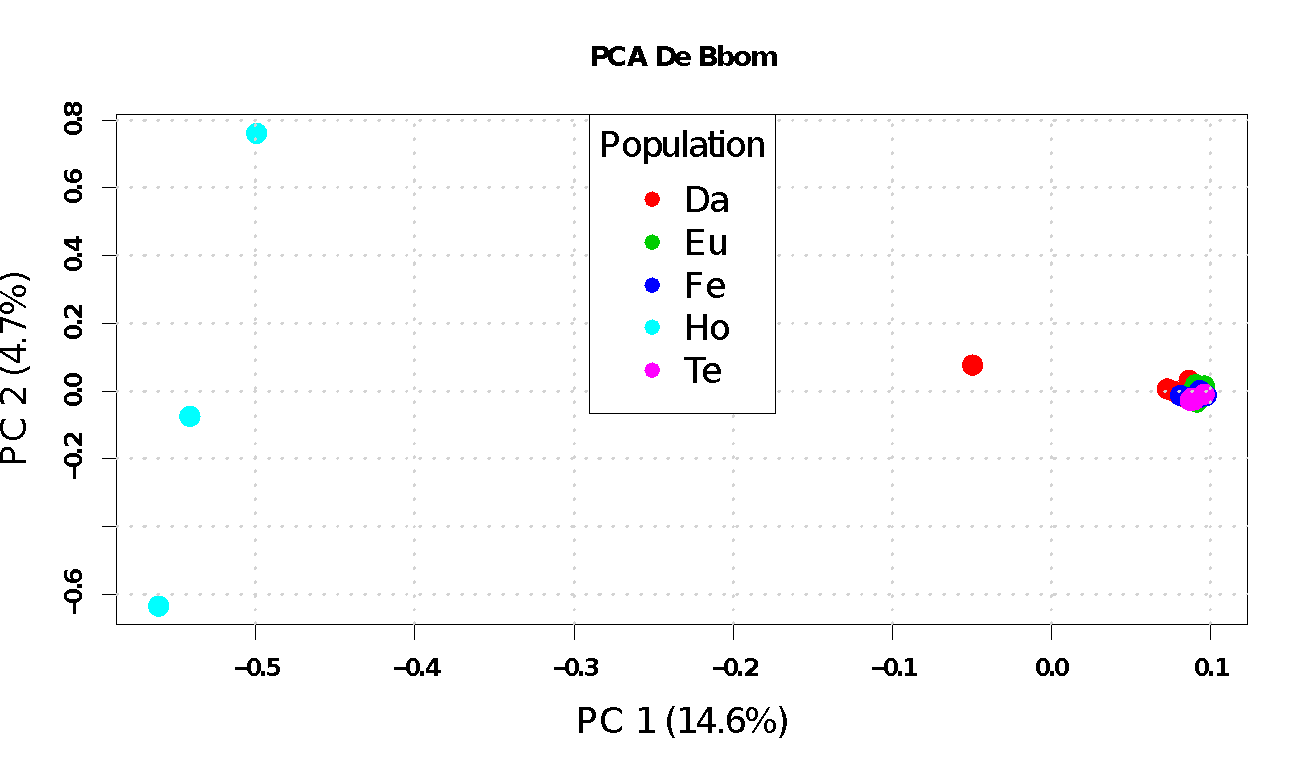


**Figure S3:** Transcriptome-wide Principal Component Analysis of 27 *Bombina bombina* tadpole specimen from four locations in northern Germany using the identity by state (IBS) method


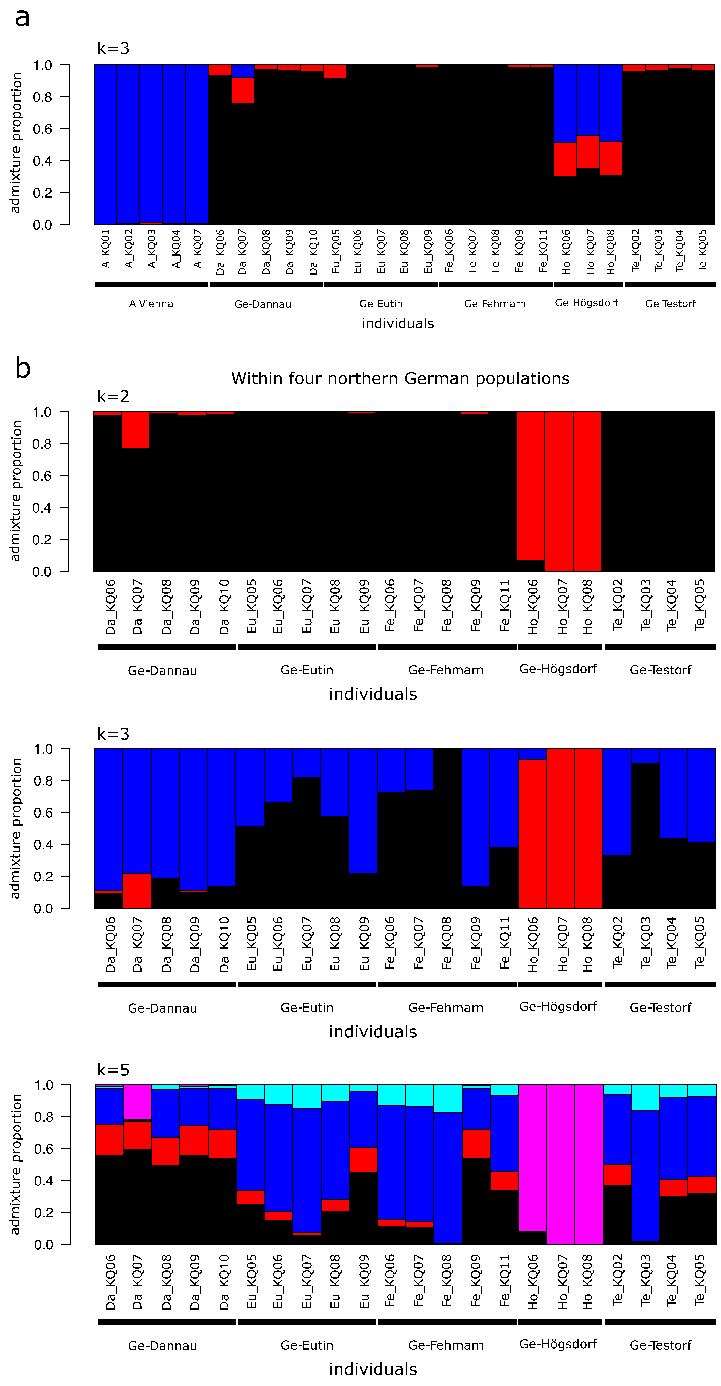


**Figure S4:** Comparison of admixture structure analysis plots produced with the software PCangsd for a) four German and one Austrian population with k=3, and for b) four German populations with k=2, k=3 and k=5

**
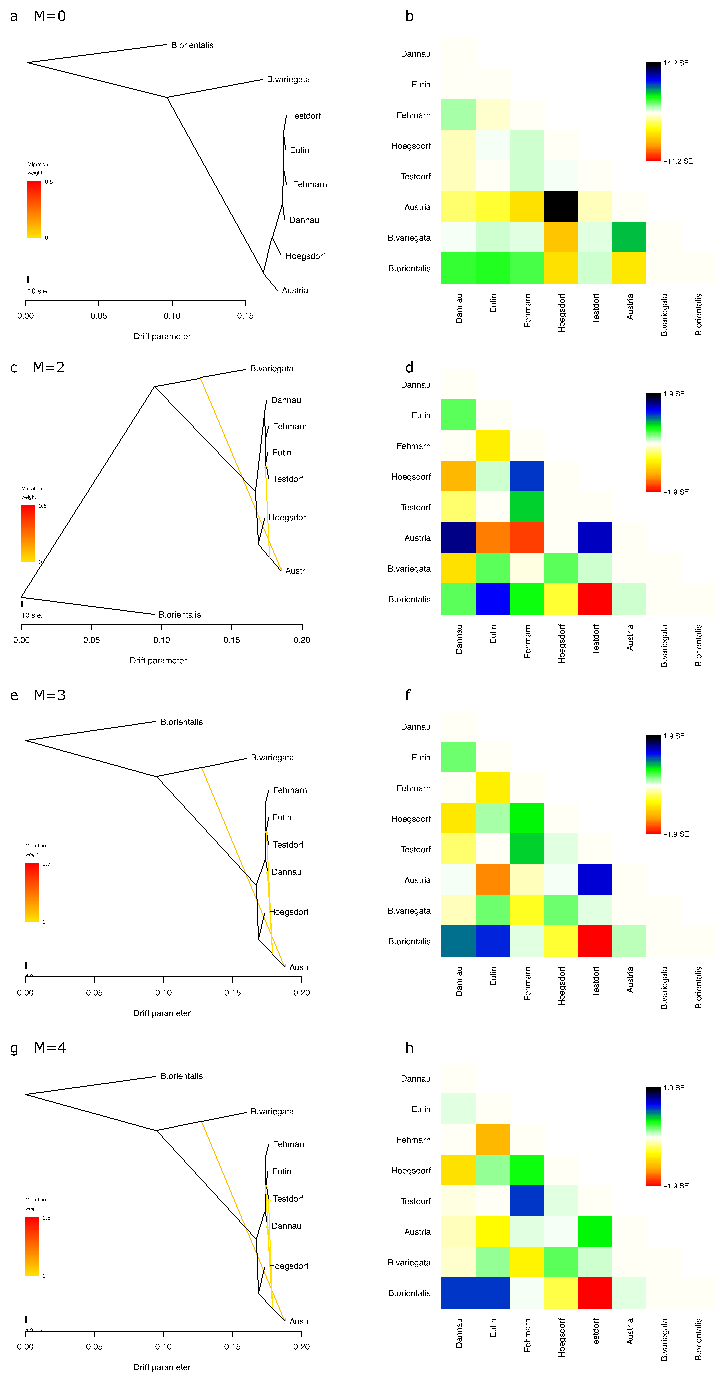
**

**Figure S5:** Treemix trees for different migration edges. *B. orientalis* is specified as root (a,c,e,g). The respective residue matrices are on the right (b,d,f,h).


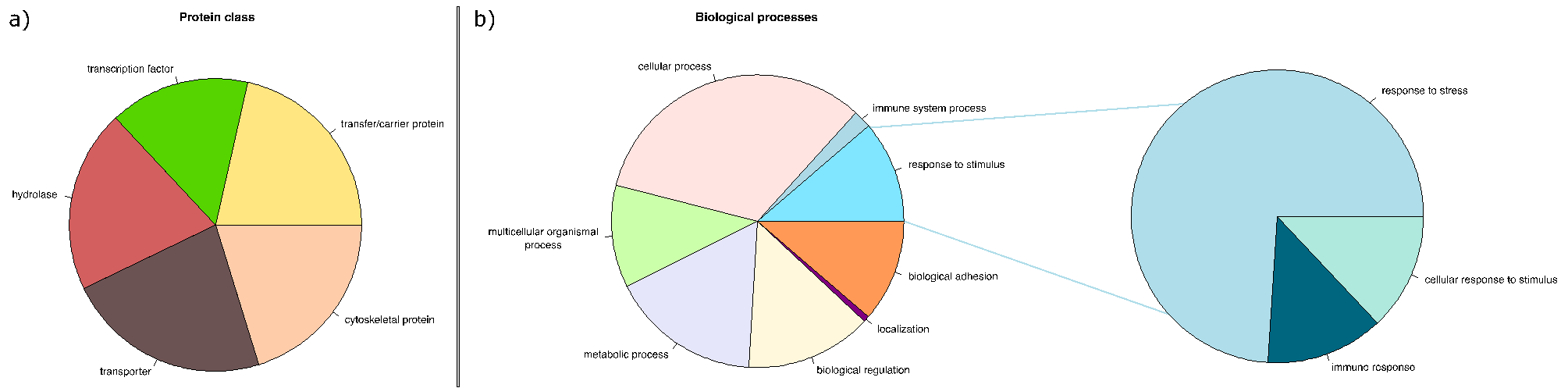


**Figure S6:** Classified gene ontology hits for five out of the seven identified genes, which introgressed from translocated Austrian toads, using the online software tool PANTHER (**P**rotein **AN**alysis **TH**rough **E**volutionary **R**elationships) Classification System v14.1. a) Classification into protein classes with five hits b) Classification into biological processes according to the gene’s involvement in pathways with a particular focus on the category “response to stimuli”.
